# Early and late Iron supplementation for low birth weight infants: a meta-analysis

**DOI:** 10.1186/s13052-015-0121-y

**Published:** 2015-03-14

**Authors:** Hong-Xing Jin, Rong-Shan Wang, Shu-Jun Chen, Ai-Ping Wang, Xi-Yong Liu

**Affiliations:** Yiwu Maternity and Child Care Hospital, No.320 Nanmen Street, Yiwu, 32200 Zhejiang China

**Keywords:** Iron, Supplementation, Low birth weight, Infant, Meta-analysis

## Abstract

**Background:**

Iron deficiency in infancy is associated with a range of clinical and developmentally important issues. Currently, it is unclear what is the optimal timing to administer prophylactic enteral iron supplementation in preterm and very low birth weight infants. The objective of this meta-analysis was to evaluate early compared with late iron supplementation in low birth weight infants.

**Methods:**

PubMed and Cochrane Library databases were searched up to May 10, 2014 for studies that compared the benefit of early and late iron supplementation in infants of low birth weight. Sensitivity analysis was carried out using the leave one-out approach and the quality of the included data was assessed.

**Results:**

The data base search and detailed review identified four studies that were included in the meta-analysis. The number of included patients was 246 (n = 121 for early supplementation and n = 125 for late supplementation) and the majority were premature infants. Across studies, early supplementation ranged from as early as enteral feeding was tolerated to 3 weeks, and late supplementation ranged from 4 weeks to about 60 days. Early treatment was associated with significantly smaller decreases in serum ferritin and hemoglobin levels (P < 0.001). In addition, the rate of blood transfusions was lower with early compared with late iron supplementation (P = 0.022). There was no difference between early and late supplementation in the number of patients with nectorizing enteroclitis (>bell stage 2) (P = 0.646). Sensitivity analysis indicated no one study overly influenced the findings and that the data was reliable.

**Conclusion:**

In conclusion, early iron supplementation resulted in less a decrease in serum ferritin and hemoglobin levels in infants with low birth rate. However, caution should be used when treating infants with iron so as not to result in iron overload and possibly negative long-term effects on neurodevelopment.

## Introduction

Iron is an essential nutrient and plays a key role in many processes including growth and development. Iron deficiency in infancy is associated with a range of clinical and developmentally important issues including neurodevelopmental deficits, delayed maturation of the auditory brainstem response, and abnormalities of memory and behavior [1,2]. Iron deficiency is estimated to range between 25% and 80% in preterms during infancy [3,4]. Low birth weight infants are particularly susceptible to developing iron deficiency anemia since they typically have small iron stores at birth and a greater need for iron due to the rapid increase in red cell mass [5-7]. Other factors that may impact development of iron deficiency anemia in low birth weight infants are preterm birth, maternal conditions (such as diabetes mellitus, hypertension, smoking, etc.) increased hemolysis, reduced red blood cell life span, low circulating erythropoietin levels, blood sampling, and loss of blood due to surgery [1,3,8].

A number of studies have found that iron supplementation increases the levels of hematologic indicators or iron status and reduces the frequency of anemia or iron deficiency in low birth weight or premature infants [8,9]. One concern with iron supplementation is that free ferrous iron may increase oxidative stress via the production of free radicals [6]. Hence, it is important to prevent not only iron deficiency but also iron overload. Currently, it is unclear at what time to administer prophylactic iron supplementation in preterm very low birth weight infants [10]. In fact, the different international associations recommend different timings for initiation of iron supplementation for these babies [10]. European Society for Paediatric Gastroenterology, Hepatology, and Nutrition Committee on Nutrition recommends prophylactic enteral iron supplementation (given as a separate iron supplement, in preterm formula or in fortified human milk) should be started at 2 to 6 weeks of age (2–4 weeks in extremely-low-birth weight infants) [11]. The Canadian Pediatric Society suggests for infants with low birth weight (<1000 g) they should receive a total intake of 3–4 mg/kg per day starting at 6–8 weeks after birth [12]. The American Academy of Pediatric recommend that that a preterm infant who is fed milk should receive a supplement of elemental iron at 2 mg/kg per day starting by 1 month of age and extending through 12 months of age [13]. The objective was to gain insight into the importance of timing of iron supplementation by evaluating early compared with late iron supplementation in low birth weight infants.

## Methods

### Search strategy

PubMed and Cochrane Library databases were searched up to May 10, 2014 for studies that compared the benefit of early and late iron supplementation in infants of low birth weight. Search terms included premature birth, preterm birth, premature infant, preterm infant, low birth weight infant, iron supplementation, early, and late. The search also included: (premature birth OR preterm birth OR low birth weight) AND (iron deficiency OR iron supplement OR iron). Included studies were randomized controlled prospective trials whose patient population were low birth weight infants (<2500 g) or premature infants (gestational age <37 weeks). Eligible studies used an intervention that involved iron supplementation (any kind), and had to compare the effect of early and late iron supplementation. Early supplementation was considered between 2–3 weeks postnatal age and late supplementation was defined as >4 weeks postnatal age. Studies were included if they evaluated infants who could tolerate enteral feeding (usually >2 weeks of age). Studies were excluded if they were single arm and did not evaluate serum ferritin and hemoglobin levels. Studies were also excluded if the intervention was combined with erythropoietin treatment, andif they were non-English, case reports, letters, or comments.

### Data extraction

The following data was extracted: author’s first name, study design, inclusion criteria, iron source, early or late supplementation, dosing route, number of subjects, gestational age, birth weight, gender, and time of evaluation. Also the level of serum ferritin (ng/mL) and hemoglobin before and after treatment was extracted as was the percent of subjects requiring blood transfusions and necrotizing enterocolitis (>bell stage 2). Two reviewers extracted the studies, and a third reviewer was consulted to resolve any disagreements.

### Quality assessment

Quality assessment of the included studies was based on Cochrane handbook version 5.1.0 Chapter 8 “Assessing risk of bias in included studies” table for validity assessment of eligible trials [14].

### Statistical analysis

The changes in serum ferritin and hemoglobin levels, blood transfusion rate, and necrotizing enterocolitis rate were compared between participants who received early supplementation and participants who received late supplementation. For the continuous data, changes in serum ferritin levels and hemoglobin levels, the data were represented as mean ± standard deviation (SD) or mean (range: min, max) for a given group in each study. The effect size was calculated as difference in means of outcomes after iron supplementation between early and late groups with estimated 95% confidence intervals (95% CI) and corresponding P values. For those data with mean (range), the SD was utilized according to the equation SD = Range/4before analysis [15]. The difference in means of outcomes greater than 0 indicated the late group was favored. The difference in means of outcomes lower than 0 indicated the early group was favored. If the means difference was zero than both the early and late groups had similar change in outcomes. For blood transfusion and necrotizing enterocolitis rates, data were represented as events/total participants for a given group in each study.

The effect size was calculated as odd ratio (OR) of outcomes after iron supplementation with estimated 95% CI between early and late groups and corresponding P values. OR >1 indicates late group had higher rate than early group; OR <1 indicates late group had lower rate than early group; OR = 0 indicated both groups had similar rate. A χ2 based test of homogeneity was performed using Cochran’s Q statistic and I^2^. I^2^ indicates the percentage of the total variability in effect estimates among trials due to heterogeneity rather than chance. Random-effects model of analysis (DerSimonian–Laird method) was used if heterogeneity was detected (I^2^ >50% or P-value <0.05). Otherwise, fixed-effects model (Mantel-Haenszel method) was used. For evaluation, a combined difference in mean with 95% CI for continuous outcomes and OR (95% CI) for categorical variables were calculated for the pooled study results. A two-sided P value <.05 was taken to indicate statistical significance for one comparison group over the other. Sensitivity analysis was carried out for the outcomes using the leave-one-out approach. Publication bias was not performed because more than five studies are required to detect funnel plot asymmetry [16]. All analyses were performed using Comprehensive Meta-Analysis statistical software, version 2.0 (Biostat, Englewood, NJ).

## Results

**Figure 1:**
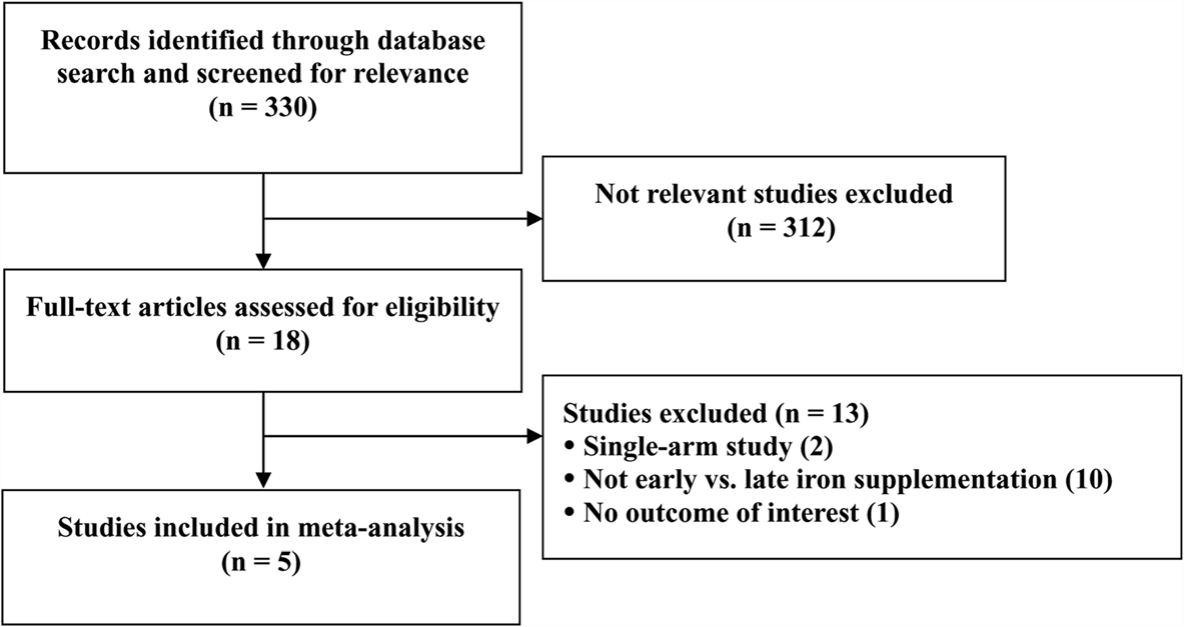
**Flow diagram of study selection.**

The search of the databases identified 330 studies of with 312 were excluded as not being relevant (Figure 1). Eighteen studies were evaluated in detail and 13 were excluded due to being a single arm study (n = 2), did not compare early versus late iron supplementation (n = 10), or did not report an outcome of interest (n = 1). Five studies were identified and their data extracted [17-20] However, the study of Franz et al. [21] was not included in the meta-analysis due to lack of detail in sample size for the outcomes evaluated. Hence, only four studies were used for the meta-analysis [17-20].

### Study characteristics

All the studies included babiess with low birth weight with premature babies being the most common (Table 1). The mean gestational age of participants (ranged from 26.7 to 32.4 weeks) was similar between studies and between groups within studies. The source of iron included colloidal iron, iron trivalent (III)-hydroxide polymaltose complex, ferrous sulfate, and ferrous succinate. The number of participants in each study ranged from 15 to 105 (n = 226) for the early supplementation group and from 13 to 99 (n = 224) for the late supplementation group. About 50% of the infants were male and the time of evaluation ranged from 1–2 days to 6 months. Early treatment ranged from as early as enteral feeding was tolerated [21] to 2 [17-19] or 3 weeks [20]. Late supplementation ranged from 4 weeks [17] to about 60 days [18,20,21].

**Table 1:** **Summary of basic characteristics of selected studies**

**Study #**	**1st AU (year)**	**Study design**	**Inclusion criteria**	**Medical iron source**	**Group**	**Route ; dose**	**Number of subject**	**Gestation age (weeks)**	**Birth weight (kg)**	**Gender (male/female)**	**Evaluated time point**
1	Joy [17]	RCT	Intramural preterm (<37 weeks gestational age) VLBW infants (birth weight 1000–1500 g) who reached full enteral feeds of 180 mL/kg/day by 2 weeks postnatal age	colloidal ferric hydroxide	early	PO; 2 mg/kg/day start at 2 weeks postnatal age	52	32.4 ± 1.7	1.35 ± 0.15	27/23	12 weeks
					late	PO; 2 mg/kg/day start at 6 weeks postnatal age	52	32.4 ± 1.6	1.33 ± 0.14	24/26	
2	Sankar [18]	RCT	Preterm VLBW (<1500 g) infants who reached at least 100 mL/kg/day of oral feeds by day 14	colloidal iron	early	PO; 3–4 mg/kg/d at 2 weeks	22	32.4 ± 1.8	1.189 ± 0.228	12/10	60 days
					late	PO; no iron until 60 days	24	31.5 ± 2.6	1.213 ± 0.196	12/12	
3	Arnon [19]	RCT	All infants with a gestational age of 32 weeks who were fed human milk and reached enteral intake of 100 mL/kg/d	Iron trivalent (III)– hydroxide polymaltose complex	early	PO; 5 mg/kg/d enteral iron polymaltose complex at 2 weeks	32	30 (27, 32)†	1.248 (0.859, 1.960)†	18/12	8 weeks
					late	PO; 5 mg/kg/d enteral iron polymaltose complex at 4 weeks	36	29 (27, 32)†	1.072 (0.830, 2.173)†	14/16	
4	Franz [21]	RCT	All inborn infants with a birth weight of < 1301 g	ferrous sulfate	early	PO; 2–4 mg/kg/d oral iron once enteral feeding was tolerated	105	26.7 (23, 33)*	0.868 (0.380, 1.300)*	na	61 days
					late	PO; started at 61 days of life at a dose of 2 mg/kg/day.	99	26.9 (23, 35)*	0.872 (0.370, 1.300)*		
5	Jansson [20]	RCT	LBW infants with a birth weight ≦2000 g and/or a gestational age of ≦35 weeks	ferrous succinate	early	PO; 2–3 mg/kg/day from 3 weeks of age	15	All: 34 (29–37)*	1.855 ± 0.430	na	1-2 days; 8–10 weeks; 6 months
					late	PO; 2–3 mg/kg/day from 2 months of age	13		1.779 ± 0.327		

Gestational age and birth weight were presented as mean ± SD.

*mean (range); †median (range).

na, not available; PO, per os;RCT, randomized clinical trial; VLBW, very low birth weight.

Generally, across all studies serum ferritin and hemoglobin numerically decreased following either early or late iron supplementation (Table 2). In the different studies the serum ferritin was measured by enzyme immunoassay and the method sensitivity ranged from 1–2.5 ng/ml and specificity of about 100%. Hemoglobin was estimated by Coulter LH 750 analyzer and the specificity and sensitivity for this assay are not reported. The percent of patients requiring blood transfusions was numerically higher for those receiving late iron supplementation compared to those receiving early supplementation. Necrotizing enterocolitis was similar between early and late iron supplementation.

**Table 2:** **Summary of outcomes of selected studies**

				**Serum ferritin (ng/mL)**		**Hemoglobin (g/dL)**			
**Study #**	**1st AU (year)**	**Group**	**number of evaluated subject**	**before**	**after**	**before**	**after**	**Blood transfusion (%)**	**Necrotizing enterocolitis (>bell stage 2)**
1	Joy [17]	early	46	112 ± 5 at 2 weeks of age	82 ± 5	12.9 ± 0.8 at 2 weeks of age	10.1 ± 0.4	2 (4.3%)	3/46
		late	47	113 ± 6 at 2 weeks of age	63 ± 3	13.1 ± 0.6 at 2 weeks of age	9.2 ± 0.4	7 (14.8%)	4/47
2	Sankar [18]	early	22	55.7 ± 12.1 at enrollment	50.8 ± 11.5	na	na	2 (9.5%)	1/21
		late	24	59.0 ± 12.1 at enrollment	45.3 ± 11.9			3 (13.0%)	0/23
3	Arnon [19]	early	32	94 ± 27 at 2 weeks of age	46 ± 20 at 8 weeks of age	12.5 ± 0.9 at 2 weeks of age	9.0 ± 1 at 8 weeks of age	1/33 (2.8%)	1/32
		late	36	90 ± 21 at 2 weeks of age	30 ± 12 at 8 weeks of age	11.9 ± 1.3 at 2 weeks of age	7.4 ± 0.7 at 8 weeks of age	10/46 (21.3%)	3/36
4	Franz [21]	early	68	85.5 (18, 282) *at birth	87.8 (9, 478)*	na	na	41/105 (39.0%)	6/105
		late	65	77.9 (13, 354)*at birth	74.2 (9, 682)*			53/99 (53.5%)	8/99
5	Jansson [20]	early	15	102 (34–220)*at 1–2 days of age	56 (16–175)* at 8–10 weeks of age 26 (18–45)* at 6 months of age	20.0 ± 2.7 at birth	10.3 ± 1.0 at 8–10 weeks of age; 11.5 ± 0.7 at 6 monthsof age	na	na
		late	13	1-2 days: 100 (45–200)*at 1–2 days of age	72 (21–170)* at 8–10 weeks of age 28 (10–115)* at 6 months of age	19.1 ± 2.9 at birth	9.7 ± 1.0 at 8–10 weeks of age 11.5 ± 0.5 at 6 months of age		

*mean(range).

na, not available.

### Quality assessment

Overall the studies were of high quality. In three of the studies the participants and personnel were not blinded to treatment (Table 3). The remaining two studies did not describe blinding. Jansson et al. [20] did not describe if allocation was concealed or if outcome assessments were blinded. Only three of the studies describe that they included an intention-to-treat analysis.

**Table 3:** **Quality assessment**

**1st AU (year)**	**Random sequence generation**	**Allocation concealment**	**Blinding of participants and personnel**	**Blinding of outcome assessment**	**Incomplete outcome data**	**Selective reporting**	**Did the analysis include an intention-to-treat analysis?**
Joy [17]	Y	Y	N	Y	Y	Y	NA
Sankar [18]	Y	Y	N	Y	Y	Y	Y
Arnon [19]	Y	NA	NA	Y	Y	Y	NA
Franz [21]	Y	Y	N	N	Y	Y	Y
Jansson [20]	Y	NA	NA	NA	Y	Y	Y

NA: not available; N: no;Y: yes.

### Serum ferritin level

**Figure 2:**
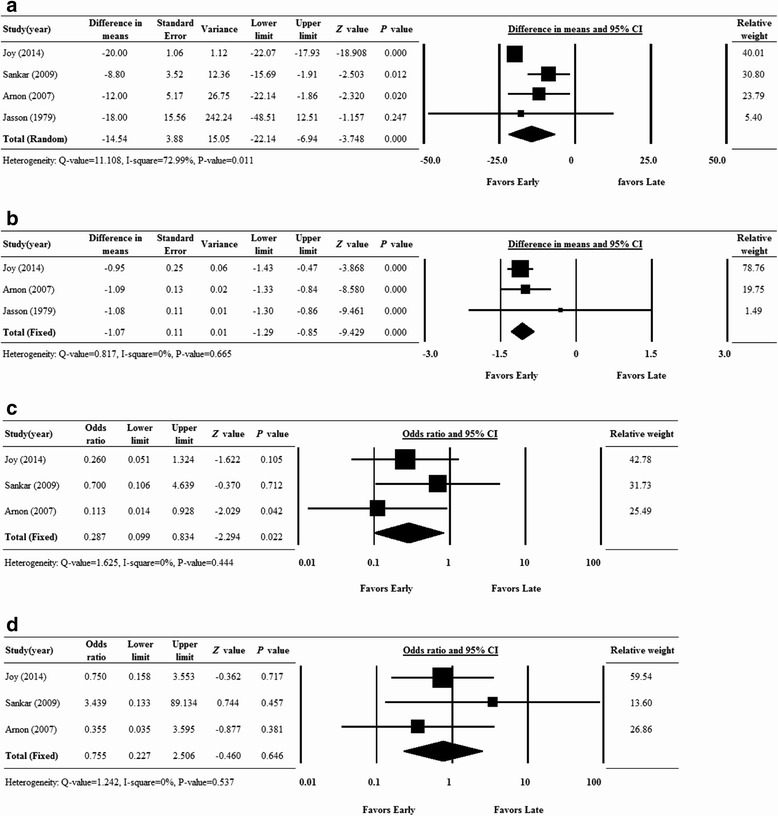
**Forest plot evaluating the serum ferritin level (A), hemoglobin level (B), blood transfusion rate (C) and necrotizing enterocolitis rate (D) of participants receiving iron supplementation were represented.** Abbreviations: CI, confidence interval; Lower limit, lower bound of the 95% CI; Upper limit, upper bound of the 95% CI.

Four studies with serum ferritin data were included in the analysis [14-17]. A random effects analysis was applied because there was evidence of heterogeneity among the studies (Q statistic = 11.108, I^2^ = 72.99%, P = .011). The summarized difference in means of change of serum ferritin levels after iron supplementation significantly favored the early iron supplementary group (difference in means = −14.54, 95% CI = −22.14 to −6.94, P <.001) (Figure 2A). The magnitude of fall in serum ferritin level was smaller in the early compared with the late iron supplementation group.

### Hemoglobin level

Three studies had complete hemoglobin data and were included in the analysis A fixed effects analysis was applied because there was no evidence of heterogeneity among the studies (Q statistic = 0.817, I^2^ = 0%, P = .665). Similar to ferritin levels, the summarized difference in means of change of hemoglobin levels after iron supplementation favored the early iron supplementation group (difference in means = −1.07, 95% CI = −1.29 to −0.85, P < .001) (Figure 2B). The decrease in hemoglobin levels was smaller in the early compared with the late iron supplementation group.

### Blood transfusion

Three studies were included in the analysis since they had complete data for the frequency of blood transfusions. A fixed effects analysis was used because there was no evidence of heterogeneity among the studies (Q statistic = 1.625, I^2^ = 0%, P = .444). The summarized OR = 0.287 with a 95% CI = 0.099 to 0.834 indicating that early iron supplementation lowered the frequency of blood transfusion rate compared with late supplementation (P = .022) (Figure 2C).

### Necrotizing enterocolitis (> bell stage 2)

Three studies that had complete necrotizing enterocolitis data were included in the analysis. A fixed effects analysis was applied as there was no evidence of heterogeneity among the studies (Q statistic = 1.242, I^2^ = 0%, P = .537). The summarized OR = 0.755 with a 95% CI = 0.227 to 2.506 indicated there was no difference between early and late supplementation in the frequency of necrotizing enterocolitis(P = .646) (Figure 2D).

### Sensitivity analysis

**Figure 3:**
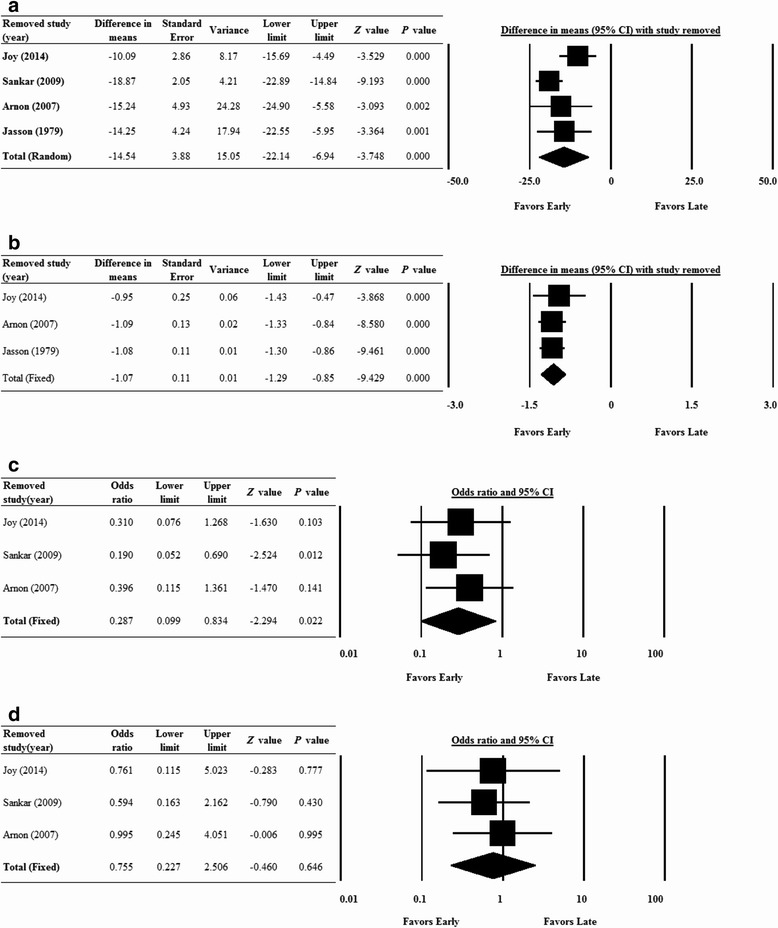
**Sensitivity analysis of the influence of each study on the pooled estimate for serum ferritin level (A), hemoglobin level (B), blood transfusion rate (C) and necrotizing enterocolitis rate (D) of participants receiving iron supplementation were represented.** The leave-one-out approach was used. Abbreviations: CI, confidence interval; Lower limit, lower bound of the 95% CI; Upper limit, upper bound of the 95% CI.

We performed sensitivity analysis where the data was reevaluated after each study was removed in turn. The direction and magnitude of the combined estimates did not change markedly for ferritin levels (Figure 3A), hemoglobin levels (Figure 3B), frequency of blood transfusions (Figure 3C), and the rate of developing necrotizing enterocolitis (Figure 3D) when any one study was removed. These findings indicate that the meta-analysis had good reliability and that no one study overly influenced the results.

## Discussion

Our analysis investigated the relative benefit of early versus late iron supplementation in low birth weight infants including premature infants. Early treatment was associated with significantly smaller decreases in serum ferritin and hemoglobin levels (P <.001). In addition, the rate of blood transfusions was lower with early compared with late iron supplementation (P = .022). There was no difference between early and late supplementation in the number of patients with nectorizing enteroclitis (P = .646). Sensitivity analysis indicated no one study overly influenced the findings and that the data was reliable.

Our study is consistent with findings that suggest early compared with later iron supplementation may benefit infants with low birth weights. The study of Lundstrom et al. [5] found that in low birth weight infants (1,000 to 2,000 gm) (N = 117) that those infants who did not receive iron supplementation (2 mg of iron/kg/day starting at 2 weeks of age) had a higher tendency (about 80% of infants by 6 months) to develop iron deficiency compared to those infants who did receive iron supplementation (about 5-10% by 6 months) [5]. Hall et al. [22] performed a randomized controlled study that compared iron nutritional status in premature infants with birth weight <1800 g (N = 20) who received iron (1.7 mg/L, 3 mg/L or 15 mg/L) added to premature formula that was fed to the infants during initial hospitalization. They found that the higher iron supplementation (3 mg/L and 15 mg/L) added at this early timepoint resulted in a reduced frequency of anemia and low transferrin saturation compared with the infants who were given the 1.7 mg/L iron supplementation [22]. A study by Miller at al. [23] found that there was no difference in conventional measures of iron status in preterm babies (24–32 weeks of gestation) who either did or did not receive iron supplementation (3–12 mg/kg/day) when the supplementation occurred relatively late (ie, 7- to 60-days after birth).

Iron deficiency in infancy is associated with growth and neurodevelopmental deficits. Steinmacher et al. [24] in a follow-up study of a prior randomized trial examined whether early compared with late iron supplementation improved neurocognitive and motor development in preterm infants (<1301 gm) [24]. The original study found that early enteral iron supplementation (as early at enteral feeding was possible) compared with late supplementation (Day 61 of life) reduced the frequency of blood transfusions and the incidence of iron deficiency in low birth weight infants [21]. In the follow-up study, they used the Kaufmann Assessment Battery for Children and the Gross Motor Function Classification Scale to evaluate neurocognitive and psychomotor development in children at 5.3 years’ corrected age who had been treated with early or late iron supplementation in the original study. The Kaufman Assessment Battery for Children is a standardized test that assesses intelligence and achievement in children aged two years, six months to 12 years, six months. The Gross Motor Function Classification Scale is a scale from 0 to 5 that classifies the severity of motor involvement of children on the basis of their functional abilities and their need for assistive technology. Steinmacher et al. found that early enteral iron supplementation compared with late supplementation was associated with a trend for better long-term neurocognitive and psychomotor development; about 19% of the early and 35% of the late supplementation group had abnormal neurological development and approximately 66% and 54%, respectively, were without disability. Gross Motor Function Classification Scale score >1 was found in 2% of patients for the early and 7% of patients in the late iron supplementation group. A limitation of the study was that the original study was not powered to evaluate neurocognitive development.

Two other studies also evaluated the use of iron supplementation in low birth weight infants on cognitive and neurodevelopment outcomes [25,26]. Friel et al. [26] investigated the effect of increased iron intake on hematologic and cognitive status in infants with low birth rate (N = 58). They evaluated two levels of iron supplementation: 13.4 mg iron/L and 20.7 mg iron/L. They found that hemoglobin and Griffith’s Developmental Assessment were not different between treatment groups (P values <.05). They did find that the number of respiratory tract infections was higher in the high compared with low iron groups, possibly indicating a detriment for administering high levels of iron. These findings suggest there is no advantage to administering elevated iron to infants with low birth weights.

Ohls et al. [25] assessed the effect of supplementing erythropoietin and iron compared with iron alone on long-term developmental outcomes in extremely low birth weight infants (≤1000-gm birth weight) (N = 172). Approximately 5 mg/kg of iron was included in both treatment groups. The study evaluated the need for transfusions, anthropometric measurements, post-discharge events, and developmental outcomes at 18 to 22 months’ corrected age. They found no significant difference in weight, length, or head circumferance between the erythropoietin plus iron compared with iron alone groups. There was also no difference between groups in rate or rehospitalization, transfusions after discharge, the percentage of patients with Bayley-II Mental Developmental Index <70 (34% for erythropoietin plus iron and 36% for iron alone) or other neurodevelopmental and cognitive functions. The authors conclude that early treatment with erythropoietin plus iron was not of greater developmental benefit than iron alone.

Our meta-analysis only included four studies and the studies were heterogenous with respect to dosage and timing of iron supplementation. For example, the definition of “early” and “late” supplementation varied across studies. Heterogeneity in studies investigating the effect of iron supplementation in low birth infants has been noted before [9] and indicates the need for more consistent designs among studies that investigate this issue. In addition, the iron sources used across the studies differed and had different oral absorption and gastrointestinal tolerance [27], which may have confounded our findings. We also did not evaluate the effect of early versus late iron supplementation on neurodevelopment, cognitive function, or growth. Further studies are required to address these important medical questions. Our findings showed that the early group had a better iron status than late group, which suggest that the prevalence of iron deficiency and iron deficiency anemia may be lower in early supplement group. However, only one included study [21] evaluated iron deficiency, hence, we were not able to analyze this directly. In addition, we did not assess how dose and a more precise evaluation of timing affected the results. This was not possible as the doses across the studies overlapped and the timing was variables. Moreover, only five studies were included in the analysis making it not practical to do subgroup analysis.

In conclusion, early iron supplementation improved serum ferritin and hemoglobin levels in infants with low birth rate. However, caution should be used when treating infants with iron so as not to result in iron overload and possibly negative long-term effects on neurodevelopment [6].

## Abbreviations

CI: Confidence level
OR: Odd ratio
SD: Standard deviation
